# Asynchronous online focus groups for research with people living with amyotrophic lateral sclerosis and family caregivers: usefulness, acceptability and lessons learned

**DOI:** 10.1186/s12874-023-02051-y

**Published:** 2023-10-06

**Authors:** Shelagh K. Genuis, Westerly Luth, Garnette Weber, Tania Bubela, Wendy S. Johnston

**Affiliations:** 1 Division of Neurology, Department of Medicine, University of Alberta, 7-123 Clinical Sciences Building, Edmonton, AB T6G 2B7 Canada; 2 itracks, Saskatoon, SK Canada; 3 Faculty of Health Sciences, Simon Fraser University, Blusson Hall 11328 8888 University Drive, Burnaby, BC V5A 1S6 Canada

**Keywords:** Amyotrophic lateral sclerosis, Online focus groups, Research design, Patients, Family caregivers, Research participation

## Abstract

**Background:**

People with amyotrophic lateral sclerosis (ALS) face disability- and travel-related barriers to research participation. We investigate the usefulness and acceptability of asynchronous, online focus groups (AOFGs) for research involving people affected by ALS (patients and family caregivers) and outline lessons learned.

**Methods:**

The ALS Talk Project, consisting of seven AOFGs and 100 participants affected by ALS, provided context for this investigation. Hosted on the secure itracks Board™ platform, participants interacted in a threaded web forum structure. Moderators posted weekly discussion questions and facilitated discussion. Data pertaining to methodology, participant interaction and experience, and moderator technique were analyzed using itracks and NVivo 12 analytics (quantitative) and conventional content analysis and the constant-comparative approach (qualitative).

**Results:**

There was active engagement within groups, with post lengths averaging 111.48 words and a complex network of branching interactions between participants. One third of participant responses included individual reflections without further interaction. Participants affirmed their co-group members, offered practical advice, and discussed shared and differing perspectives. Moderators responded to all posts, indicating presence and probing answers. AOFGs facilitated qualitative and quantitative data-gathering and flexible response to unanticipated events. Although total participation fell below 50% after 10–12 weeks, participants valued interacting with peers in an inclusive, confidential forum. Participants used a variety of personal devices, browsers, and operating systems when interacting on the online platform.

**Conclusions:**

This methodological examination of AOFGs for patient-centred investigations involving people affected by ALS demonstrates their usefulness and acceptability, and advances knowledge of online research methodologies. Lessons learned include: early identification of research goals and participant needs is critical to selecting an AOFG platform; although duration longer than 10–12 weeks may be burdensome in this population, participants were positive about AOFGs; AOFGs offer real world flexibility enabling response to research challenges and opportunities; and, AOGFs can effectively foster safe spaces for sharing personal perspectives and discussing sensitive topics. With moderators playing an important role in fostering engagement, AOFGs facilitated rich data gathering and promoted reciprocity by fostering the exchange of ideas and interaction between peers. Findings may have implications for research involving other neurologically impaired and/or medically vulnerable populations.

**Supplementary Information:**

The online version contains supplementary material available at 10.1186/s12874-023-02051-y.

## Introduction

With increasing use of online research methods, asynchronous online focus groups (AOFGs) have emerged as a distinct, digital method that allows research participation at times and from locations that are convenient for participants [1–3]. AOFGs have been advocated for hard-to-reach populations, including those experiencing a range of physical disabilities [1, 4–6], using augmentative and alternative communication aids (AAC) [7–9], and/or living in rural or remote locations [5, 10–12]. They reduce research participation barriers for people with disabilities by providing time to consider and input responses using standard technology or AAC [2, 5, 11, 13] and by alleviating the immediacy and response rate expected in synchronous, face-to-face communication [9]. Further, this online method promotes a safe environment conducive to the investigation of potentially sensitive topics [14–16]. Some studies suggest that AOFGs may result in reduced relational satisfaction and group interaction [1, 3, 17]. However, others indicate that AOFGs generate similar thematic content to face-to-face focus groups [13, 18, 19], while facilitating individual expression, reflection on others’ experiences, and discussion of different or shared perspectives [14, 20, 21]. Active moderation has been identified as critical to encouraging constructive discussion [2, 13, 14], however there has been limited examination of moderators’ activities within AOFGs.

In this investigation, we describe the use of AOFGs in a study involving a medically vulnerable population – people living with amyotrophic lateral sclerosis (ALS) – and their family caregivers (collectively, people affected by ALS). ALS is a multisystem neurodegenerative disease with a median overall survival of 30 months after symptom onset [22]. Characterized by rapidly progressive motor impairment, people with ALS (PwALS) face severe disability and eventual respiratory failure [23]. Dysphagia and/or dysarthria is experienced at disease onset by 25%-30% of patients [24], and almost all PwALS experience functional communication challenges with disease progression [25, 26]. Similar to other medically vulnerable or fragile populations [4, 6, 27], PwALS face research participation barriers arising from complex medical needs, disabilities, and rapid disease progression [28–32]. Their caregivers face barriers related to time-consuming, caregiving responsibilities [5, 17, 33]. ALS is also considered a ‘rare disease’ [23]. This restricts local recruitment [34], while those living distant from research centres face disability-related travel barriers [28, 31].

We found three AOFG studies with participating PwALS [7, 35, 36]. Each of these studies involved a single group with less than 10 participants. Study duration varied from 3 to 8 weeks. While most health studies have sample sizes less than 15 [4, 27, 37, 38], AOFG sample sizes commonly vary from 10 to 30 [13]. Groups typically run for less than seven days but may extend for longer periods [3, 38].

To date, ALS research using AOFGs has focused on study findings [7, 35, 36]. Here we report on methodological lessons from the ALS Talk Project (ALS Talk) and explore participants’ use of technology and interactions within the AOFGs. We analyze qualitative data to better understand interaction between participants, participant experience with AOFGs, and moderator technique. We also describe design flexibility. Specifically, we ask: (1) Are AOFGs a useful and acceptable research method for investigations involving people affected by ALS? And, (2) What can be learned from the methods used in ALS Talk that might be useful for other investigations involving PwALS and caregivers?

## Methods

ALS Talk was approved by the University of Alberta’s Research Ethics Board (Pro0008471). Two amendments were approved: the addition of questions pertaining to the COVID-19 pandemic (approved March 20, 2020), and an optional topic exploring participation in observational research and data sharing (approved April 21, 2020). All participants provided informed consent.

### Context

ALS Talk provided context for this study. Grounded by a patient-oriented research approach [39], ALS Talk investigated health communication throughout the disease course from the perspective of PwALS and family caregivers [40]. We selected AOFGs for their potential to answer our research questions by facilitating discussion of diverse individual experiences, as well as providing opportunities for consensus development [21]. They also circumvent participation barriers and foster a supportive environment for people affected by ALS [41, 42]. A four-member participant advisory council [43] and collaborators at ALS multidisciplinary clinics provided input on and beta-tested the discussion guide on the itracks Board™ platform (itracks) platform. We structured discussion topics sequentially, focusing on health communication from the beginning of the diagnostic journey to end of life. Topics included information seeking, research participation, and improving ALS communication. Participants discussed past, current, and anticipated health communication experiences and/or needs. PwALS and caregiver discussion guides included parallel questions with adaptations to account for perspective. We summarize methods for thematic analysis of ALS Talk data elsewhere [40, 44, 45]. Here we analyze unpublished data about the usefulness and acceptability of AOFGs for people affected by ALS.

### Participants and recruitment

To achieve a national, geographically diverse sample, we purposively recruited PwALS and caregivers from Canada’s largest provinces (British Columbia (BC), Alberta (AB), Ontario (ON), and Quebec (QC)) and two small provinces (New Brunswick (NB) and Nova Scotia (NS)). We recruited participants with a formal ALS diagnosis [46] and family members who presently or previously cared for a PwALS. PwALS/caregiver dyads were not required. Participants were required to be over 18 years of age, able to communicate in English, and have internet access. All qualifying volunteers were invited to participate.

We recruited via multidisciplinary ALS clinics, the Canadian Neuromuscular Disease Registry (CNDR) [47], and regional and national non-profit ALS Societies. Local research staff recruited at multidisciplinary clinics. CNDR sent mass mailouts to eligible patients in their registry. ALS Societies recruited through local ‘community leads’ and through online newsletters and/or social media. We recruited between July 2019 and January 2020, seeking 15 participants per focus group. During this period, we intermittently contacted recruited participants by email to provide study updates. Because attrition is a common challenge for AOFGs [14, 28, 30], we over-recruited where possible.

PwALS and caregivers participated in separate focus groups, thus facilitating interaction between peers [37]. We conducted focus groups for PwALS and for caregivers in BC, AB, and ON. Due to low enrollment in QC, NB, and NS, PwALS from these provinces participated in one combined focus group; there were insufficient numbers for a caregiver focus group from these provinces.

There was no interaction between the seven focus groups. Four AOFGs started on January 7th, 2020 (AB, ON) and three on March 11th, 2020 (BC, QC/NB/NS). Data collection occurred January to July 2020.

### Technology and platform

AOFGs were hosted on the secure itracks platform [48]. itracks met our aims through its capacity to meet institutional ethics requirements, accommodate a range of communication modalities, host both open- (qualitative) and closed-ended (quantitative) questions, capture longitudinal, asynchronous data for the duration of the project, provide automatically generated transcripts and MP3 recordings of audio or MP4 video data, facilitate both individual and group email notifications, and accommodate a range of electronic devices. Further, this platform met expectations for researcher support in setting up and managing the AOFGs, and participant usability.

itracks uses a threaded web discussion board format. Participants entered text (via typing or inputs supported by participant device capabilities including audio transcription and eye-gaze software), video, and/or audio responses. Responses were viewed by group co-members in the input modality used by the author. People could participate in the AOFGs at their convenience from any location with internet access. Participation was possible via an internet browser on a Windows-based PC or MacOS computer, IOS or Android tablet, or smartphone. A mobile app allowed participants to download questions and respond without internet access. Saved responses automatically uploaded to the software database when the device next connected to the internet.

We provided participants with step-by-step, illustrated guidance for platform registration and setting up personal profiles. We provided technical assistance by email, telephone, and in-person when requested and as possible. The itracks help-desk was also available for assistance with platform onboarding and ongoing technical support, including live chat, toll-free telephone contact, and email. The platform included safety enhancing features. Participants used secure authentication to login to the platform. Study data were encrypted in transit and stored on secure encrypted servers. Participants could answer questions or communicate with the moderators confidentially using itracks’ privacy mode. We posted contact information for a selected list of ALS support services, mental health supports, and the project email on the platform. Participants were reminded of these supportive resources prior to discussions pertaining to end of life.

### AOFG design and procedures

**Fig. 1:**
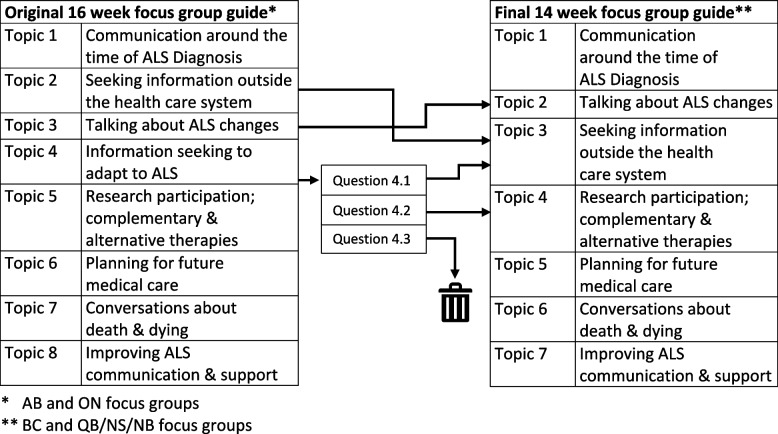
Focus group guides, topic consolidation

Based on validated interview guides from a pilot project [49], we developed an eight-topic/16-week discussion guide. We incorporated feedback from our participant advisory council and collaborators following discussion guide beta-testing. We used the eight-topic guide for the groups in AB and ON. After noting data redundancies suggestive of saturation [50] for questions about information seeking and receiving participant feedback about perceived overlap between questions, we consolidated topics to create a seven-topic/14-week guide for the BC and QC/NB/NS focus groups. Questions were restructured to retain data integrity across focus groups (Fig. 1). One question yielding little relevant data was dropped.

Before beginning the discussion questions, participants completed optional demographics and support questionnaires. They read guidelines for group interaction and confidentiality, and were asked to introduce themselves and interact informally within their group. Following this ‘getting started’ week, we introduced discussion topics biweekly. Topic specific question threads were posted each week. In the consolidated seven-topic discussion guide, twenty-four question threads contained open-ended questions without words restrictions. Closed-ended questions included multiple response/multiple-choice (*n* = 4), single response/multiple-choice (*n* = 1), ranking exercise (*n* = 1), and Likert scales (*n* = 1). Closed-ended questions were accompanied by open-ended questions inviting further details or explanations. After participants posted answers to closed-ended questions, graphical summaries of quantitative question data were available for viewing and discussion. Weekly question posts included one to five sub-questions, sequenced to yield richer data within the targeted discussion area [51] (Additional file 1). All questions were optional. Participants were required to post an initial response to question threads before they could read and respond to other participants. We encouraged participants to post responses at least weekly. Questions remained open for the duration of the AOFG allowing participants to add further details or ‘catch up’ if needed.

AOFGs were moderated by research associates with expertise in patient-oriented research and qualitative research methods (SKG, WL). A moderator read and responded to participant posts twice each weekday, and once each day on weekends. Moderators were guided by the following goals: stimulate further input, clarify meaning, and/or encourage group interaction. Each discussion topic was actively moderated for its two-week duration. Participants received group emails introducing weekly discussion questions, noting potentially sensitive material within questions, and encouraging participation. We sent email reminders to participants who had not engaged with the focus groups for 2 to 3 weeks. We repeated reminders three times if we did not hear back from absent participants.

Two optional topics were added while the AOFGs were underway. The first, pertaining to COVID-19 health communication, was posted on March 21st, 2020 (AB, ON) and April 3rd, 2020 (BC, QC/NB/NS). It was available concurrently with scheduled discussion topics. Second, we invited participation in a two-week discussion of ALS observational research and data sharing following completion of the scheduled topics. We kept detailed fieldnotes throughout the study.

### Analysis

itracks automatically generated transcripts from text-based AOFG discussions. A professional transcriptionist transcribed videorecording. These latter transcripts were verified. There were no audio-only data. All transcripts were uploaded into NVivo 12 ™ software. itracks and NVivo 12 analytics facilitated analysis of quantitative data, for example, word counts, number of participant posts, and analysis of closed-ended questions. We used NVivo 12 to facilitate organization, identification of themes, and coding of qualitative data.

Guided by our research questions, we coded data on interaction between participants and participant experience with AOFGs into individual parent nodes. We used NVivo 12 to auto-code moderator comments into a parent node. These data formed our dataset for qualitative analysis.

Following close reading of these data a codebook was developed (SKG, WL) and reviewed by subject experts (WSJ, TB). Qualitative data were analyzed using conventional content analysis [52] and the constant-comparative approach [53] during codebook development and coder training. Coders received training on 10% of the data and discrepancies were resolved through discussion to consensus during the training period. Coders independently coded another 10% of data to assess intercoder reliability, which achieved a Kappa coefficient of 0.96. Descriptive statistics were used to summarize participant characteristics. We do not include numeric data when reporting the results of qualitative analysis. Descriptions of design flexibility were based on fieldnotes.

Analytic rigour was enhanced by a robust sample size [13]. We took the following measures to strengthen credibility and dependability: prolonged engagement, debriefing with subject and clinical experts, and documentation of all research stages [54–56]. Thick description and exemplars are provided to improve transferability of qualitative findings [55].

## Results

### Participant characteristics

One hundred people participated in the AOFGs (Table 1). Caregivers included partners, spouses, siblings, and adult children. PwALS reported being 1 to 29 years post-diagnosis (mean = 3.96; median = 3), with three (6%) being 10 + years post-diagnosis. We did not collect data quantifying physical disability or dysarthria. However, qualitative data indicated that participants were navigating a range of functional disabilities, with some experiencing relative independence while others were physically dependant and/or reliant on AAC.

**Table 1:** Characteristics of ALS Talk focus group participants

Characteristic	PwALS (*n* = 51)		Caregiver (*n* = 49)		Total (*n* = 100)
**Gender**					
Female	19	37%	38	78%	57
Male	31	61%	8	16%	39
No response	1	2%	3	6%	4
**Province**					
Alberta	15	29%	17	35%	32
British Columbia	10	20%	16	33%	26
Ontario	17	33%	16	33%	33
Quebec/New Brunswick/Nova Scotia	9	18%	0	0%	9
**Rural/Urban**					
Rural or Small town^a^	12	24%	14	29%	26
Urban center	38	75%	33	67%	71
No response	1	2%	2	4%	3
**Age**					
18–29	0	0%	3	6%	3
30–39	1	2%	3	6%	4
40–49	4	8%	10	20%	14
50–59	10	20%	15	31%	25
60–69	21	41%	10	20%	31
70 +	14	27%	5	10%	19
No response	1	2%	3	6%	4
**Educational level**					
Completed graduate studies or MD	9	18%	10	20%	19
Completed undergraduate degree	13	25%	15	31%	28
Completed technical program or apprenticeship	17	33%	14	29%	31
Completed high school	11	22%	8	16%	19
No response	1	2%	2	4%	3
**Onset site (of self or family member with ALS)**					
Limb	34	67%	31	63%	65
Bulbar	10	20%	8	16%	18
Don't know	3	6%	5	10%	8
Other	2	4%	2	4%	4
Prefer not to answer	1	2%	1	2%	2
No response	1	2%	2	4%	3
**Time since diagnosis (of self or family member with ALS)**					
1 year	9	18%	11	22%	20
2 years	15	29%	12	24%	27
3 years	11	22%	9	18%	20
4 years	5	10%	8	16%	13
5 years	4	8%	3	6%	7
6 years	2	4%	1	2%	3
7 years	1	2%	1	2%	2
8 years	0	0%	1	2%	1
11 + years	3	6%	0	0%	3
Prefer not to answer	0	0%	1	2%	1
No response	1	2%	2	4%	3

^a^Population under 10,000

### Technology

Participants primarily contributed to AOFGs using text modalities. Some participants mentioned using eye-gaze and/or other assistive technologies, however, the platform did not track AAC use. Two participants posted video responses (16.48 min in total). Audio-only modalities were not used. Most participants used desktop computers; 18% used smartphones and 4% used tablets. Participants used a range of devices, browsers, and operating systems (Additional file 2).

Participants tended to contact the research team rather than itracks for technical support. Onboarding challenges included incompatibility between the mobile app and personal devices, problems navigating the mobile app, and using an incorrect project code or log-in link. During the project some participants reported losing messages before they were posted, unexpected platform downtime, technical difficulties with the tablet app, difficulty scrolling through long discussions, and challenges navigating between questions and topics.

### Participation

Multidisciplinary clinics and non-profit ALS Societies played key roles in recruitment. Recruitment was most effective when potential participants were contacted individually by telephone, email, or face-to-face, rather than mass mail-outs or social media. Only two people were recruited via ALS Society online newsletters and/or social media. Five people responded to CNDR’s mass mailed (*n* = 96) or emailed (*n* = 46) study information (Table 2). Of the 137 people recruited, five participated in interviews since there were insufficient numbers for an AOFG in their region. These data are not included in this investigation. Thirty-two individuals did not follow through from recruitment to self-introduction on itracks. Those who provided reasons for dropping out during this period reported changed life circumstances, disease progression, and/or technical challenges.

**Table 2:** ALS Talk recruitment

	**Multidisciplinary Clinics**^a^	**ALS Societies**	**CNDR**	**Word of mouth**^b^	**Unknown/other**	**Total**
BC	12	19	n/a	1	1	33
AB	33	0	2	4	0	39
ON	17	23	1	0	3	44
QC	10	0	0	0	0	10
NB/NS	7	2	2	0	0	11
Total	79	44	5	5	4	

^a^One clinic recruited in BC, QC, and NB. Two clinics recruited in AB and ON

^b^Heard about the study from a PwALS or family caregiver and contacted the research team

**Fig. 2:**
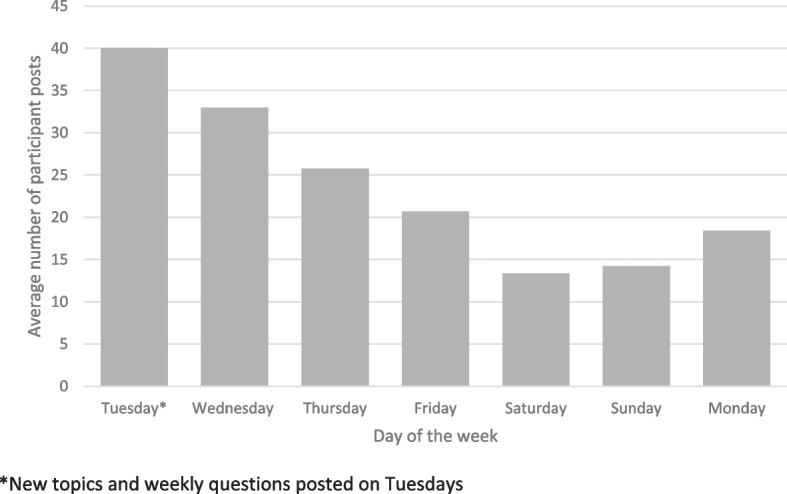
Average participant messages per weekday over the course of the focus groups

All seven AOFGs experienced attrition over time with participation falling below 50% overall during Topic 6. This occurred after 10 (BC and QC/NB/NS groups) and 12 (AB and ON groups) weeks of participation (Table 3). Twenty-two individuals provided reasons for discontinuance: disease progression and/or death (*n* = 10), time constraints (*n* = 6), technical challenges (*n* = 5), and emotional stress (*n* = 1). In the two groups beginning with < 15 members, the BC PwALS group maintained robust discussions, whereas the QC/NB/NS PwALS group experienced rapid attrition. We posted new topics and weekly discussion questions on Tuesday mornings. The majority of responses and interaction occurred over the first four days (Fig. 2).

**Table 3:** Participation in ALS Talk by topic

	**Recruited**	**Completed introduction on platform**	**1.Communication around the time of ALS diagnosis**	**2.Talking about ALS changes**	**3.Looking for information outside health care system**	**4.Research, & complementary or alternative therapies**	**5.Planning for future medical care**	**6.Death and dying**	**7. Improving ALS communication**	**Optional topic: COVID-19**	**Optional topic: Observational Research & Data sharing**
**Alberta**	**39**	**32**	**29**	**23**	**28**	**24**	**16**	**12**	**11**	**13**	**9**
PwALS	20	15	14	12	14	12	10	8	7*	9*	6*
Caregiver	19	17	15	11	14	12	6*	4*	4*	4*	3*
**British Columbia**	**33**	**26**	**24**	**20**	**21**	**19**	**19**	**17**	**15**	**16**	**14**
PwALS	14	10	9	9	9	9	9	8	7	9	6
Caregiver	19	16	15	11	12	10	10	9	8*	7*	8*
**Ontario**	**44**	**33**	**27**	**25**	**26**	**22**	**20**	**17**	**15**	**14**	**9**
PwALS	22	17	14	13	14	12	12	10	9	9	6*
Caregiver	22	16	13	12	12	10	8*	7*	6*	5*	3*
**QC/NB/NS**	**21**	**9**	**8**	**7**	**6**	**4**	**3**	**3**	**2**	**0**	**n/a**
PwALS	14	9	8	7	6	4*	3*	3*	2*	0*	n/a
Caregiver	7	n/a	n/a	n/a	n/a	n/a	n/a	n/a	n/a	n/a	n/a
**Total**	**137**	**100**	**88**	**75**	**81**	**69**	**58**	**49**	**43**	**43**	**32**

^*^ ≤ 50% participation in AOFG based on number of people who completed introductions on the platform

### Engagement within AOFGs

**Fig. 3:**
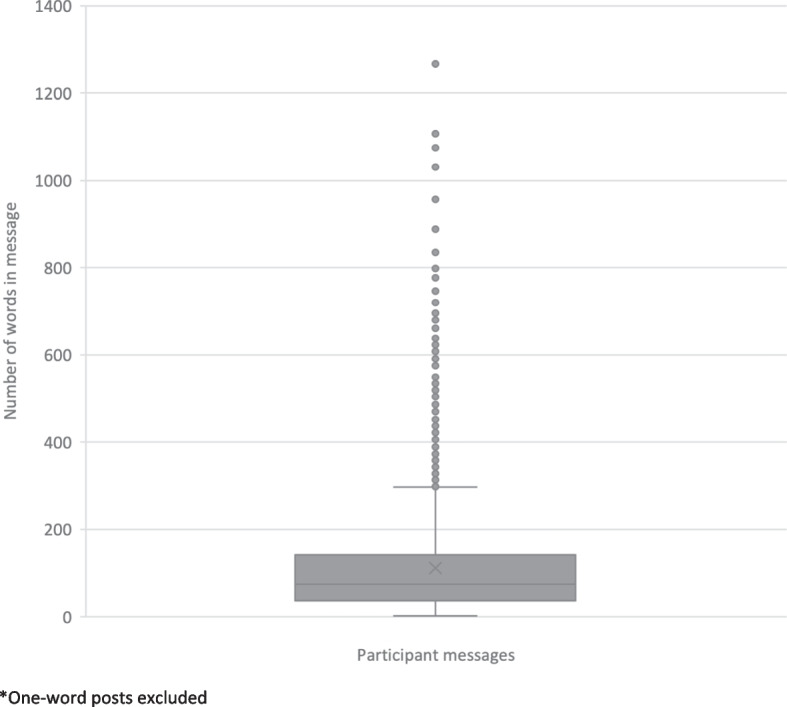
Distribution of participants’ responses by word count

We examined engagement by analyzing the length of participant posts by word count, and the number and length of conversation threads within discussion topics. We excluded one-word posts and the ‘getting started’ week from our analyses. These posts (for example, “thanks”) were most frequently used to acknowledge the completion of quantitative exercises. The mean post length was 111.48 words (SD = 11.47). Figure 3 plots the distribution of participants’ posts by word count. Sequentially threaded posts indicate participant engagement with discussion questions, moderators, and/or other participants. Conversation threads varied from single to 12 posts in response to discussion questions (Table 4).

**Table 4:** Examples of different length threads

Number of posts in thread	Illustrative examples
1 post (*n* = 341)	1. *“We first heard about edavarone on the news—there was a special about how a couple travelled to Japan and I clearly remember seeing the woman who had ALS talk about how, since getting this treatment, she was able to open a vitamin/medication bottle. It was incredible and we were so excited. This led us to research edavarone and get all the information about the research and the treatment available.”* (P54, Caregiver)
2 posts (*n* = 806)	1. *“Hey P4 [PwALS], I saw a shout out for you from ALS Canada on Twitter in recognition of your volunteer service. Well done and thank you from one pALS to another.”* (P68, PwALS) 2. *“Thanks, P68 [PwALS]!”* (P4, PwALS)
3 posts (*n* = 189)	1. *“We did talk about financial support, but there is not a lot of support in that area for us. We have talked to social work about living wills, advance directives, and end of life decisions, but haven't done it yet.”* (P9, Caregiver) 2. *“Hi P9 [Caregiver], you looked for a lot of information online! Did the information you found answer your questions? What sorts of websites did you prefer to gather information from? Did you talk to anyone on the health care team about how to approach financial support?”* (Moderator) 3. *“Definitely the ALS Society of [province] and Canada. I sought out support groups on facebook and followed people on instagram. Looked at some medical journals. I have talked about financial support a few times, but apparently we don't qualify for any assistance because I "make too much money." Which is still frustrating.”* (P9, Caregiver)
4 + posts (*n* = 819)	1. *“The first [ALS] symptoms I presented was when I was exercising at the gym….Now one year had transpired since the episode in the gym. At no time was ALS mentioned and I certainly was not aware of even what ALS was…The original neurologist then referred me to the [multidisciplinary clinic] for further examination. This was about 16 months from my initial symptoms. It was at this examination that I was told my diagnosis was ALS…What I find disheartening is the length of time from the diagnosis and the fact that no health care professionals during the first year and a half ever once mentioned ALS. I lost this time where I could have been developing an earlier plan to deal with this disgusting disease.”* (P73, PwALS) 2. *“Hi P73 [PwALS], you certainly experienced a difficult journey to this diagnosis. And, I’m with you, it is an unspeakably terrible disease. Do you wish that a health professional had mentioned the possibility of ALS (or of a serious problem) earlier? Or been more open about what they were looking for during the various tests? Does this experience influence your trust in health professionals or your trust that they will tell you what you need to know?”* (Moderator) 3. *“When I think back there were opportunities to tell me about a possible ALS diagnosis. Especially from the neurologist I first saw and the neurosurgeon. In retrospect I feel they both suspected ALS. I am the first patient with ALS for my family doctor so I can see why she didn’t go down that road. What she did do was minimize my symptoms. That didn’t impress me. Yes, I wish they were more open about the testing. I even encouraged a dialogue during the nerve testing but there was no mention of possible ALS. And yes, it has negatively influenced my trust in Doctors.”* (P68, PwALS) 4. *“Thank-you for your very authentic answer. ALS is uncommon and very scary (that's an understatement, I know). Nonetheless, many people want to understand what the doctor is exploring. It sounds like you were really searching for some expert input and communication. How about other people in the group: Do you think that a health professional should let you know if there is a possibility of ALS, or of a serious problem?”* (Moderator) 5. *"Yes, I think you should be told. You need time to process and prepare.” (P44, PwALS)*

There were 1041 threads consisting of a participant response to posted discussion questions, followed by interactive posts between the participant and moderators, and/or other participants. There were 523 threads consisting of participant response to discussion questions without further interaction, despite moderator prompting in 88.7% (*n* = 464) of these threads.

**Fig. 4:**
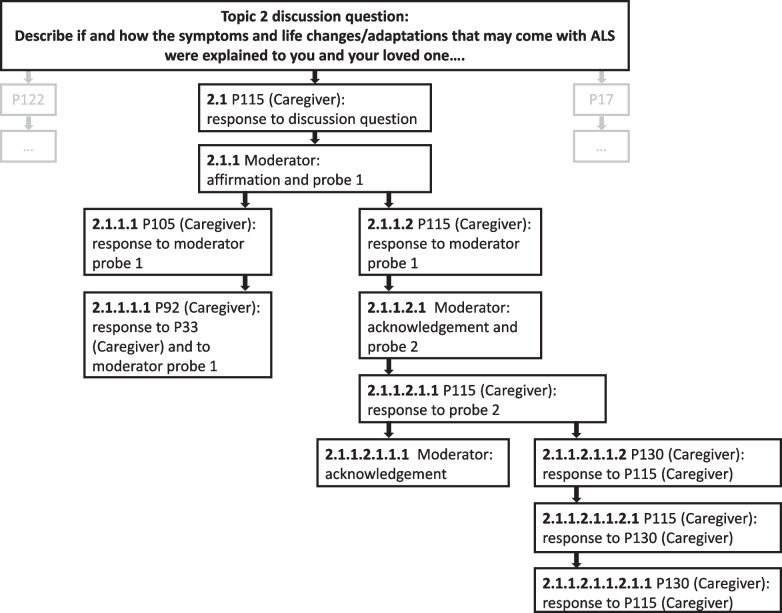
Illustration of branching discussion threads

Engagement within AOFGs was also indicated by networks of branching interactions (Fig. 4). Participants initiated 67.7% (*n* = 400) of the 591 branching conversation threads, which varied in length from 1 to 10 posts.

The platform’s privacy mode was used in 25 conversation threads: moderators initiated 15 private threads, in 12 cases initiating privacy mode in response to a participant’s public post, and participants initiated 10. Twelve threads included confidential and/or sensitive subjects. Eight included discussions of AOFG management, for example assistance with question navigation, interceding to protect confidentiality, and notification of potentially sensitive topics. Three threads included a personalized ‘thank-you’ to highly engaged participants. And three threads involved moderator-facilitated communication to assist participants exchanging contact information and/or information tangential to discussion topics.

### Themes exemplifying interaction between participants

Three primary themes exemplify the interaction between AOFG participants. First, participants made supportive or affirming statements. These were frequently followed by sharing related experiences or information. Second, participants offered practical advice. Finally, participants expanded on thoughts and perspectives shared by others, thus ‘developing ideas together.’ This occurred in single responses or in dialogue between participants (Table 5).

**Table 5:** Engagement between participants, qualitative themes

Themes	Illustrative quotations
Support and affirmation	• *“That is really awesome that your [sibling] was willing to voice bank on your behalf! It is a lot of work, but personally I am glad that I did it*.” (P33, PwALS)
Practical advice	• *“If I may, I would suggest you reach out to the ALS Society now, rather than wait. Some of the support takes time and there are waiting lists. An example is self-managed care funding. There is a 12–18 month waiting list once the lengthy application is received. Often to receive services or equipment an OT [occupational therapist] must do an assessment and make the request on your behalf.”* (P20, Caregiver)
Developing ideas together	• *“I would like to add to P24’s [PwALS] comments RE mental health issues…health professionals at the ALS clinic could have a bigger role in addressing psychological issues. A simple question to open the discussion could be: How did you take the news of being diagnosed with ALS and how your family and you are managing? How and what can we do to help you now… Also, regular assessment should be done at the clinic to make sure that individuals have the necessary strength and resources to cope, regardless of where they are in their ALS journey.”* (P47, PwALS)
	• *“I completely agree P52 [Caregiver] about transparency with tests. While it may be a scary reality for the patients, I think that being able to access test scores to compare/set realistic expectations would be helpful. My [PwALS] is trying to live in the moment and not dwell on what is to come. Unfortunately, that has led to most major changes becoming urgent matters.”* (P59, Caregiver) *“P59# [Caregiver], I have gone so far as to take screen shots of breathing test scores to check and to compare to past tests. Like your [PwALS], my [PwALS] likes to live in the moment. When the progression is quicker, I can see how this may lead to dealing with things as they become urgent. There is such a fine balance between living with independence and accepting help before it is needed.*” (P52, Caregiver) *“P52 [Caregiver], there are times when I feel like this disease isn't moving fast and we get into a routine with our 'new normal.' Then, seemingly overnight, everything changes. I am constantly on edge just waiting for the next emergency…You are right, it is definitely a fine balance!”* (P59, Caregiver)

### Moderating

We analyzed moderator posts and found two primary themes: ‘I hear you’ responses signaling active presence, and specific probing strategies. Moderators also personalized interactions by referring to participants by name.

‘I hear you’ responses included positive affirmations, neutral acknowledgements, empathetic responses, and off-topic replies. This latter category included, for example, responses to participants who mentioned family or vacations. Specific probing strategies included repeating participants’ words and asking for clarification and/or further information, and summarizing to confirm meaning and/or improve understanding of participants’ perspective. Moderators also encouraged discussion of different or shared viewpoints by referring to participants’ posts and inviting input from other group members (Table 6). Frequently moderators used multiple strategies:

*Hi P114 [PwALS], You’ve provided a great list of what a “good explanation” should look like! Very helpful to hear your thoughts in detail.* [‘I hear you’, affirmation]

*It seems like you value an explanation of the facts in layman’s terms, followed by an opportunity for more in-depth discussion of the why’s, attention to enough information (not too much/not too little), and also information in written form that can be taken away and read. Reasons should be given for recommendations. Have I captured your thoughts with this summary?* [Probing strategy, summarizing]

*Can you tell me a little more about what you would like to see in terms of an explanation of the “whole process of ALS”?* [Probing strategy, repeat back]

**Table 6:** Moderator posts, qualitative themes

Themes	Illustrative quotations
**I ‘hear’ you**	
Positive affirmations	• “*I’m with you both on this P68 [PwALS] & P4 [PwALS]. The ALS society is invaluable – and they certainly are filling gaps in the health care system.”*
Neutral acknowledgments	• *“Hi P122 [Caregiver], Thanks for sharing about the topics you would or wouldn’t talk to a health professional about.”*
Empathetic response	• *“I’m so sorry to hear of all these challenges that COVID has brought to your family, P92 [Caregiver]. I’m also really sorry to hear how unsupported you feel in terms of the health professionals.”*
Engaging with unrelated topics	• *“Gorgeous photo! I grew up around that area and am yearning for a visit to your part of the world. It sounds like you have an amazing pile of grand-kiddos!”*
**Specific probing strategies**	
Repeat back	• *“You note the “sharing of information” as a problem. Do you see this as a breakdown in sharing between doctors, or between you and doctors?”*
Summarizing	• *“It sounds like in response to health professionals' lack of awareness you're wondering if you should have filled that information void *via* the internet. Am I putting words into you mouth'? Or does this sound right to you?”*
Reference to other participant’s perspectives	• *“P84 [Caregiver] mentioned previously that some of the supports for his [PwALS] took a long time to get. Do you feel supported to get your team’s recommended strategies implemented in time for them to be useful to you and your [PwALS]?”*
Inviting group input	• *“I’m wondering what others in the group think: Do you think it’s helpful if doctors were more open about ALS as a potential diagnosis? Or is it better to wait until it is confirmed?”*

### Participant experience

PwALS and caregivers commented on their experience as AOFG participants. Topic 7 questions elicited more than 70% of these comments (n = 196). Participants were asked to share what they liked and/or did not like about the AOFGs, other topics they would have liked to discuss, their views on project duration, and potential improvements. Most participant comments were positive. They focused on methodological elements of the study and personal benefits including promoting reflection or conversation, learning from peers, and altruism. A few participants made constructive comments, focusing on methodological elements, particularly study length, and technical shortcomings. (Table 7).

**Table 7:** Participant experience, qualitative themes

Themes	Illustrative quotations
**Methodological elements**	
Moderator role	• *“I have appreciated the back-and-forth discussion with the reflections, especially [the moderators’] comments that tell me you have read and heard my input. I have found that sometimes the clarifying questions coming back to me stimulate me to think more.*” (P1, Caregiver)
Peer interaction	• *“The ability to see other participant's responses and sometimes get a bit of conversation going was a nice difference from other studies that were simply questionnaire or interview based.*” (P4, PwALS)
Flexibility	• *“I like the format of this focus group. The pressure of immediate response can affect the way I interact.*” (P61, PwALS) • *“This online focus group study has been much easier to do than a previous one that I did in-person. It enabled me to log on any day or time of day to reply to the questions or just read some of the other replies.*” (P78, Caregiver)
Study duration	• *“I found the study a little bit too long, with some of the topics a bit overlapping.*” (P33, PwALS) • *“[The focus group] was long, but it allowed for continuity and a sense of membership. As I read the topics, reflected on my [PwALS’] and my experiences, and figured out how to write things down in a way that made sense to others, it was good to think that I didn't have to reflect on the totality of these experiences in one long questionnaire. I think this longer, staged methodology worked well for this type of reflective input.*” (P38, Caregiver)
**Personal benefits**	
New reflections or conversations	• *“This [study] has been really good at helping me to discuss issues with my [PwALS], so we can explore the options available. We will continue to live and function, but we need to be more realistic in our future planning.*” (P91, Caregiver)
Learning from peers	• *“What I found useful for building mental fortitude is learning and sharing the stories of other people with ALS, like many in this group. When P114 [PwALS] describes how he deals with his secretions and how that battle line is advancing, I learn something about survival skills.*” (P49, PwALS)
Altruism	• *“I am sharing our experiences so the healthcare system and its professionals can see what it looks like from our vantage point. People were kind and tried their best, but I'm grateful for this study so they can learn and adapt their current practises. If our experience helps others so they can adapt and learn from our challenges, then I'm glad to share our story.*” (P124, Caregiver)
**Technology**	• *“The platform was difficult for me because I use an eye gaze computer. It’s low resolution so that meant a lot of scrolling left and right. Plus, selecting questions could be difficult because of the precision required. I also preferred, in many cases, composing my answers offline and copying and pasting it.*” (P68, PwALS) • *“It was simple to navigate, even for a computer dinosaur like me.”* (P114, PwALS)

### Design flexibility facilitated by AOFG methods

AOFG methodology facilitated design adaptations in response to unanticipated events. First, following the March 2020 declaration of the COVID-19 pandemic, we added a discussion topic exploring participants’ perceptions of and experiences with pandemic-related health communication. Thirty-one PwALS and 22 caregivers contributed to the COVID-19 data subset [45]. One participant noted, “It was good to see the flexibility when the COVID-19 topic was included” (P4, PwALS). Another reflected, “The study went longer that I expected but with COVID I had nowhere else to be” (P20, Caregiver).

Second, in response to an unanticipated need for patient-centred input on a funding application, we added an optional topic investigating perspectives on ALS observational research and data sharing. Eighteen PwALS and 14 caregivers participated [57].

Finally, we consolidated the discussion guide to address data redundancies within the study. And, in response to severe attrition in the QC/NB/NS PwALS group, we adapted this group’s schedule for posting discussion questions. This allowed the remaining participants to proceed through Topics 5, 6, and 7 at their own pace, but reduced interaction between remaining participants. To decrease participant burden, we omitted the final, optional discussion topic. Three participants contributed to Topics 5 and 6. Two contributed to Topic 7.

## Discussion

Our investigation suggests that AOFGs are a useful and acceptable approach for patient-centred investigations involving people affected by ALS. Here we discuss inclusive research design, data generated from both individuals and group discussion, and the acceptability of AOFGs. We then reflect on four lessons that may be useful for other investigations involving PwALS and caregivers. We focus on technology, participant engagement, real world flexibility, and safe and ethical research practices.

AOFGs facilitated broad inclusion of people affected by ALS, including those frequently excluded from research due to disability-related travel and other barriers [28, 31, 58]. This is a prominent challenge in Canada with its sparse, geographically dispersed population and small number of ALS research clinics, all located in large urban centres [59]. Nonetheless, whereas only 18.35% of Canadians live outside urban centres [60], AOFGs enriched our sample with a high proportion (26%) of people living in small towns and rural areas. Moreover, ALS clinical trial participants are likely to be male, younger, and have slower progression rates than the eligible ALS patient population [32]. Our sample, however, had a broad range of characteristics including age, time since diagnosis, and site of onset, allowing us to capture perspectives from a heterogenous sample of PwALS. For example, similar to other population-based ALS studies [61], our sample was weighted neither towards younger age of onset, nor long-term survivors. This suggests that AOFGs provide viable participation options for patients and caregivers navigating a range of ALS-related functional disabilities. Incorporating these methods may help address concerns about the limited generalizability of ALS research [32, 62].

AOFGs generated both individual reflections and group discussions. While individual reflections have been described as a limitation of AOFGs [17], we designed the study to allow different levels of engagement [14, 21] within topics and over time. Participants identified this flexibility as a positive feature of AOFGs. Nonetheless, the majority of participant responses occurred in interactive discussion threads. This contributed to data quality by generating further details, clarifications, and discussion of shared or contrasting perspectives. Participants also affirmed and supported other group members, potentially contributing to a safe environment for sharing sensitive information [1, 2, 63].

This study provides evidence for the acceptability of AOFGs for people affected by ALS. Similar to other populations, PwALS and caregivers appreciated the convenience and flexibility of AOFGs [2, 3, 5] and valued opportunities to interact with people experiencing similar challenges [1, 15, 27, 37]. They valued the reduced time pressure of asynchronous participation [7–9] and the option to provide considered, thoughtful responses [11, 13]. People completing the study expressed primarily positive views about their research experience. They highlighted what they had gained from participation, including benefits from interacting with peers and moderators, thus drawing attention to the importance of designing studies that ‘give back’ to participants [64, 65].

### Lesson learned 1. AOFG platform

Although online meeting platforms have proliferated in response to COVID-19 [66], meeting software is not designed for ongoing, asynchronous interactions and data collection over days or weeks. AOFG studies require a platform that is continuously available for the study duration, allowing participants to access the platform at their convenience. Early identification of an AOFG platform that meets research needs and objectives is critical for effective research planning. This includes the allocation of financial and human resources, and the timely development and beta-testing of a discussion guide.

We explored both free and commercial software. Our key goals included: continuous data capture for the project’s duration; ability to run multiple, separate AOFGS within the same platform; options for text, audio, and video input; accommodation for a range of devices; moderator control to post questions separately for each group; password-protected, invitation-only access; data storage compliant with institutional data policies; and a user-friendly interface for participants and moderators. itracks met these needs, whereas, at that time, other online meeting and discussion board platforms lacked the required accessibility and/or security features. Unanticipated but beneficial features included quantitative data collection, email broadcasts from the platform, and the capacity to generate reports, including participation statistics.

Despite increasing use of communication technology across generations [67–69] and the detailed guidance provided to participants, platform onboarding was time consuming. Once successfully onboarded, most participants successfully navigated the platform. Nonetheless, personal, knowledgeable, and compassionate technical support is critical for successful AOFGs. Similarly, technical support from the platform providers is valuable for effective study management.

### Lesson learned 2. Fostering participant engagement

Here we discuss participant engagement in relation to recruitment, retention, and moderator involvement within groups. Similar to other ALS research [28], ALS Talks’ recruitment phase was associated with attrition. AOFG recruitment was improved by individual contact in-person or by telephone or email. This highlights the importance of ‘buy-in’ across recruiting research centres and investment in human resources, and recruitment over a shorter period. Although those participating for the duration of the study were positive in their evaluation of AOFGs, attrition over the course of ALS Talk was greater than that reported for ALS clinical trials [28, 70, 71]. However, unlike clinical trials which exclude the majority of PwALS based on symptom severity and/or disease duration [32, 71], ALS Talk had broad inclusion criteria, with the majority of participants reporting three or more years since the ALS diagnosis. Based on ALS prognostic norms [23] and feedback from participants, this resulted in attrition as participants navigated challenges associated with disease progression. Although our sample remained robust for AOFG research [13], our participation rate fell below 50% during the topic “Death and dying,” which occurred weeks 11–12 (BC, QC/NB/NS) and 13–14 (AB, ON). Participation trends therefore suggest that < 10–12 weeks AOFG duration may be less burdensome in this population. However, the coinciding discussion topic may have been a confounding factor contributing to lower participation.

The literature provides little guidance for the degree or type of moderator involvement required to foster AOFG engagement and generate rich data. Our analysis suggests that specific strategies to establish moderator ‘presence’ and promote group discussion are important. Moderators should maintain a consistent pattern of interaction, logging onto the platform frequently, addressing participants by name, and actively engaging with participant posts. Moderators should strategically encourage group interaction by drawing attention to expressed perspectives or experiences and inviting input from other group members. Researchers should be aware of the time commitment and budget required.

### Lesson learned 3. Real world flexibility

AOFGs facilitated effective response to unanticipated events. When the COVID-19 restricted in-person research attendance [72], ALS Talk continued uninterrupted. Moreover, we seamlessly expanded our investigation to include health communication during a public health emergency. This not only extended our findings to emergency and disaster preparedness for PwALS and their families [45], it also demonstrated reciprocity [64] as we acknowledged and provided space for the pandemic-related concerns that were emerging organically within the AOFGs. Further, despite increasing ethical [73, 74] and practical [64, 75, 76] reasons for including patient voices in health research, garnering meaningful patient engagement prior to research funding is challenging. AOFGs facilitated nimble response to an unanticipated need for patient-centred input on observational ALS research. This data, derived from an optional ALS Talk discussion topic, informed the research protocols, patient engagement strategy, and successful funding application of CAPTURE ALS [77]. Finally, AOFGs facilitated flexible response to challenges within the study. By viewing anticipated events as opportunities, evaluating for ‘fit’ with existing research goals, and seeking appropriate ethics approval, researchers may leverage the benefits of AOFG methods.

### Lesson learned 4. Safe and ethical research practices

The challenge of maintaining anonymity and confidentiality in face-to-face focus groups [78] is magnified for rare disease populations. AOFGs gave participants’ control over the name and visual representation they used within their group, thus creating a confidential, safe space. Asynchronous participation provided time to compose responses, thus reducing the danger of unintentional disclosures [78]. Platform security features, however, included automatic logout when there was no discernible user activity for a period of time. This may have inadvertently created challenges for participants using AAC and/or requiring extra time to compose messages. Moderators also played a key role in reducing risk of harm [78, 79]. They kept participants informed at all stages of the research, read and responded as appropriate to every posted comment within 24 h, and were alert to distress or potential confidentiality breeches. Further, itracks’ privacy mode facilitated tactful intervention, such as facilitating between-participant conversations for those wishing to exchange confidential information. Despite the option to answer questions in privacy mode, the vast majority of participants discussed personal and/or potentially sensitive questions openly in their groups.

Research participation places burdens on PwALS and caregivers [28, 31]. Although altruism may motivate research participation [80], patient-oriented research emphasizes reciprocity [64, 65] – the importance of ‘giving back’ to those who participate in research. Similar to other AOFG investigations [2, 5, 37], ALS Talk participants expressed appreciation for the support received and information learned through their interactions within the AOFGs. This suggests that AOFGs can simultaneously facilitate rich data gathering and enact the ethical principle of reciprocity.

AOFGs can be designed to be safe, ethical spaces for participants to share their experiences and perspectives. Strategies to support success should include a platform with built-in safeguards for patient anonymity and confidentiality, a strong moderator presence, and attention to principles of reciprocity.

### Strengths and limitations

This study was strengthened by its investigation of data derived from a national study with seven focus groups. However, despite robust sampling and methodological and interpretive rigour, our study had practical and methodological limitations. First, participation required access to the internet and technology, as well as the ability to interact online. Despite the high proportion of Canadian internet users [81], access is inconsistently distributed across rural regions [82]. itracks’ mobile app may have provided participation options in areas with intermittent internet, however, people in areas without internet access were unable to participate. This may have influenced sampling and study results. Second, AOFGs were conducted in English. This may have influenced study findings as Canada has two official languages (English and French) and 11.9% of Canadians are conversant in French only. 1.9% are conversant in neither language [83]. Third, although attrition due to progression was anticipated, we could not account for discontinuance among those who did not volunteer a reason. This limits the conclusions we can draw about attrition in this study. Fourth, our analysis may have resulted in underestimating participant interaction. Because one-word responses were frequently used to acknowledge quantitative exercises, we used NVivo analytics to exclude one-word responses from our analysis. Some of these responses may, however, have discursive significance, for example affirming a co-participant’s statement or expressing emotion via an emoji. Finally, similar to all studies with qualitative data, our results cannot be directly generalized to other geographical jurisdictions or populations.

## Conclusion

Prompted by the COVID-19 pandemic and increasing habituation to communication technology, researchers are exploring the application of new approaches and distance technologies for ALS research [84, 85], including clinical trials [58, 86]. This study offers a methodological examination of the usefulness and acceptability of AOFGs involving people affected by ALS. It supports recent research indicating the success of at-home, ALS research facilitated by online technology [31, 58] and offers an innovative approach for gathering both qualitative and quantitative data pertaining to patient and caregiver experiences and perspectives. The discussion of ‘lessons learned’ offers practical recommendations for AOFG investigations. Findings may have implications for research involving other neurologically impaired and/or medically vulnerable populations.

Further research is needed to examine how online, asynchronous methods might be incorporated into other ALS research studies, including clinical trials. Given the increasing emphasis on patient engaged research [73, 74], for example, researchers might explore how AOFGs could be used to enhance patient engagement in research. Implementation of AOFGs will be enhanced by further investigation of online platforms, efficient models of moderation, strategies to expedite recruitment, the relationship between study duration and attrition, methods to effectively track study dropouts for longer term AOFGs, and equitable access for those less familiar with online technology.

### Supplementary Information

**Additional file 1.**

**Additional file 2.**

## Data Availability

The dataset generated and/or analysed during the current study are not publicly available due to parameters of our REB application/approval but are available from the corresponding author on reasonable request.

## Abbreviations

AAC: Augmentative and alternative communication
ALS: Amyotrophic lateral sclerosis
ALS Talk: ALS Talk Project
AOFG: Asynchronous online focus group
PwALS: Person/people with ALS
