# Supplementary material for: Asynchronous online focus groups for research with people living with amyotrophic lateral sclerosis and family caregivers: usefulness, acceptability and lessons learned
Source: BMC Med Res Methodol. 2023 Oct 6;23:222. doi: 10.1186/s12874-023-02051-y (PMC10557269; doi:10.1186/s12874-023-02051-y)
Supplement: Supplementary file 1 — Additional file 1. [file 12874_2023_2051_MOESM1_ESM.pdf]

Additional file 1 for "Asynchronous online focus groups for research with people living with amyotrophic lateral sclerosis and family caregivers: Usefulness, acceptability and lessons learned"

Shelagh K. Genuis[1], Westerly Luth[1], Garnette Weber[2], Tania Bubela[3], Wendy S. Johnston[1]*

* Correspondence: wendyj@ualberta.ca
[1]Division of Neurology, Department of Medicine, University of Alberta, Edmonton, Alberta Canada
[2]itracks, Saskatoon, Saskatchewan, Canada
[3] Faculty of Health Sciences, Simon Fraser University, Burnaby, British Columbia, Canada
Full list of author information is available at the end of the article

ALS Talk Project Discussion guide
(Asynchronous online focus groups for people living with ALS)

Formatting has been modified from the online platform for readability.

Discussion guide

1. Communication around the time of ALS diagnosis 1
2. Talking about ALS changes 2
3. Looking for information outside the health care system 4
4. Research & complementary/alternative therapies 7
5. Planning for future medical care 10
6. Conversations about death and dying 12
7. Improving ALS Communication and support 14
Optional topic: Observational research & data sharing 18
Optional topic: COVID-19 21

1. Communication around the time of ALS diagnosis

<u>**1.1** [Topic 1, week 1]</u>

Well, here we go! We're going to start by talking about communication around the time of the ALS diagnosis. We are especially interested in how you learned about ALS and your experience talking with health professionals.

After you answer the questions, you will enter the focus group. You will be able to read the comments of other focus group members. Feel free to build on other people's ideas, share your related experiences, ask each other questions, etc. Remember - the more you interact with each other, the more you will get out of the discussion!

How did you discover that you have ALS? Please share your experience - from the time of first symptoms to the ALS diagnosis.

Here are some questions to guide you as you share your experience:

- What symptoms did you have, or what happened to make you seek information or help from a health professional?
- Who did you interact with as you were diagnosed with ALS? A family doctor? A neurologist? Someone else? How was your experience different as you dealt with different health care professionals?
- Who explained ALS to you? If you can remember, what was discussed during that conversation? What aspects of your diagnosis were explained well? What was not explained well? Did you receive enough information? Too much information? Please describe.
- *At the time* you were diagnosed, what were the important things that you wanted to know?

1.2 [Topic 1, week 2]

Some really interesting reflections have been shared over the last week - thank you so much! The interaction and 'building of ideas between people' is really great too. We're going to expand our discussion with the following questions. Please share your thoughts! Feel free to add to the previous question if you wish. You will know that someone has added a comment when you see a little speech bubble icon under a question. For example, this means that there are 2 comments under Question 1 that I haven't yet read:

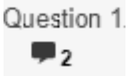

1. *As you think back* on the experience of being diagnosed with ALS, what do you _now_ wish the health care professionals had discussed with you at that time? Was there something you wanted or needed the doctor or other health professional to say or do that they did *not* say or do? Over the past week you have given some thought to your journey to the ALS diagnosis. Can you summarize the most important issues that should be discussed around the time of diagnosis?

2. There are frequently important people who share our life journeys. Was there someone with you when the health care professional explained the ALS diagnosis to you? If so, how did that person(s) participate in the conversation? What role did they play for you?

2. Talking about ALS changes

2.1 [Topic 2, week 1]

Thank-you for the great input on Discussion Topic 1. Over the next two weeks we are interested in your experiences talking to health professionals about the changes and symptoms that may

be experienced as one lives with ALS. Please share your experiences and insights! After you answer the questions, you will be able to read and explore other people's comments. Please add your thoughts to other people's ideas! Share your related or different experiences. Ask each other questions, etc. The more you interact, the more you will get out of the discussions :)

Please describe if and how the symptoms and life changes/adaptations that may come with ALS were explained to you. These might include changes to mobility, breathing, eating and/or speech. They might also include changes in day-to-day activities like getting dressed or managing technology.

Here are some questions to consider as you share your experiences:

- What was explained well? Can you describe the characteristics of good or helpful explanations about ALS changes and/or managing symptoms?
- What was *not* explained well? Can you describe characteristics of poor or unhelpful explanations about ALS changes and managing symptoms?
- Who got these conversations about managing symptoms and/or possible physical changes started?
- And, how are different health professionals helpful (or *not* helpful) as you learn about and/or help manage different ALS changes?

2.2 [Topic 2, week 1]

Health professionals have told us that finding the best time to talk about possible physical changes can be challenging. Some people want to know in advance. Some people want to talk about physical changes as they occur.

Please share your thoughts about the best time to talk about possible and/or future ALS-related changes. Here are some questions to consider as you share your thoughts:

- How prepared were you (or, how prepared *are* you) for conversations about possible changes to the way you complete day-to-day activities? Or changes to your mobility? Breathing? Eating or swallowing? Changes to your voice? Have conversations about possible future changes occurred before you felt ready to discuss these things?
- Please share your ideas about *the timing* for conversations about possible future changes. Do you wish a health care professional had talked with you earlier or later about these issues?
- Some people prepare in advance for possible changes in speech. This might include voice banking. How do you feel about taking action to prepare *in advance* for a possible change that has not yet occurred?

2.3 [Topic 2, week 2]

In previous discussions, some have touched on social and/or interpersonal life changes that ALS brings. These are important topics. Sometimes health professionals don't know how or if to talk about these changes. And sometimes they don't get it right when they do. We'd like to learn from your experiences and insights.

1. Please describe if anyone on the health care team talked to you about social or interpersonal life changes related to ALS. Did you experience social or interpersonal life changes that were *not* addressed by a health care professional and which you *wish* were discussed? Please describe.

2. Do you think the health care team *should* talk with people with ALS and their families about social and/or interpersonal life changes? Why or why *not*? If *yes*, how and when should health professionals talk about these topics?

3. Which of the changes listed below have you experienced? Mark as many answers as is appropriate for you. If we've missed something, add a note and tells us what's missing.

Which of the following changes have you experienced?
(after you make your selection, you will see the group results)

- ☐ Work status
- ☐ Your role as a breadwinner
- ☐ The tasks you do within your home
- ☐ The relationship with your spouse/partner
- ☐ Relationships within the family, for example, with children or grandchildren
- ☐ Relationships with friends and people you socialize with
- ☐ The way you socialize with others
- ☐ Your life goals and/or priorities
- ☐ What you think is important in life
- ☐ The way you think about the future
- ☐ Something else?

3. Looking for information outside the health care system

3.1 [Topic 3, week 1]

Thank-you for sharing your experiences & thoughts about communication at the time of diagnosis, and also talking about ALS changes. It's really valuable and appreciated! During the

next two weeks we're going to talk about looking for information outside the mainstream health care system.

Information about ALS can come from so many different places - websites, blogs, Facebook, the ALS Society, other people, media and more. Sometimes the information is really helpful :) Sometimes it isn't :(We're interested in learning what you knew about ALS before the ALS diagnosis, what information you found helpful or not helpful after you were diagnosed and what is important to you as you look for information about managing ALS.

This question includes a short poll! Please respond to the poll and then answer the questions in your reply.

1. What did you know about ALS before you were diagnosed? For example: Did you know anyone who had it? Had you read about it? Did you hear about the Ice Bucket Challenge and learn about it at that time?

2. Did you look for information about your symptoms or about ALS *before* the formal diagnosis by a neurologist?
    - If *NO*, please describe (in your written response) if there was a reason that you did not look for information.
    - If *YES*, **please fill in the poll below.** Select all of the places that you looked for information about ALS or your symptoms *before* you were diagnosed. In your written response, please share what questions were you trying to answer and how the information you found was helpful or *not* helpful.

If we missed something in our poll, select "other" and tell us what we missed!

Where did you look for information about ALS or about your symptoms before you were diagnosed?

(after you make your selection, you will see the group results)

- ☐ I searched for information on the internet
- ☐ I talked to other people (family, friends, acquaintances)
- ☐ I talked to someone who works in a health food store
- ☐ I talked to a naturopath or alternative health practitioner
- ☐ I found information in the media (including online news)
- ☐ I looked at social media (e.g. Facebook, Instagram, Twitter etc)
- ☐ I found information provided by an ALS Society (e.g. website, personal contact)
- ☐ Other

3.2 [Topic 3, week 1]

Did you look for additional information from sources other than a health care professional *after* you learned of the ALS diagnosis?

- If *NO*, please describe why you did not seek further information. Did you have unanswered questions after the ALS diagnosis? Was there information you still needed or wanted?
- If *YES*, what information were you looking for? What did you want to learn about? Share how the information you found was helpful and/or *un*helpful. Please use the poll below to indicate what information you were looking for. You can choose more than one answer.

Please respond to the questions after you fill in the poll below!
If we missed something in our poll, select "Something else" and tell us what information you looked for.

I looked for information about...
(after you make your selection, you will see the group results)

☐ Prognosis – the expected, average disease course for ALS
☐ Living with ALS
☐ Treatment options
☐ Clinical trials
☐ Alternative or complementary therapies or treatments
☐ CBD oil or medical marijuana
☐ The life experience of peoples who have ALS
☐ The life experience of families/caregivers of peoples with ALS
☐ Spiritual support
☐ Psychological support
☐ Financial or legal support
☐ Advance directives or 'living wills'
☐ Advanced care decisions or end-of-life decisions
☐ Something else

3.3 [Topic 3, week 2]

Please describe what makes the following information sources helpful and/or *not* helpful as you live with and manage ALS, or as you've thought about therapies/treatments. What kind of information or support are you looking for? Do you go to different sources to find different

kinds of information/support? What makes you believe (or *not* believe) the information you read or hear about?

- *Internet* - which websites? social media? blogs?
- *Other people?*
- *ALS Society?*
- *Media?*
- *Other?*

3.4 [Topic 3, week 2]

Sometimes people talk to health professionals about information they find outside the mainstream health system. Sometimes they don't.

1. Have you discussed information you found in places outside the mainstream health system with a health care professional? For example, have you talked to a health professional about information you learned from other people, or information you found online? Why or why not? If you have, how did the health care professional respond to these discussions or to your questions?
2. Could you describe if there are some topics you would talk to a health professional about, and other topics that you would *not* talk to a health professionals about?
3. How might health professionals be helpful as you explore information from outside the mainstream health system?

4. Research & complementary/alternative therapies

4.1 [Topic 4, week 1]

Many people living with ALS are aware of and interested in ALS research. You might feel really positive about research and about participating. You might find that research participation is a burden. You might be disappointed that there isn't more that you can participate in. You might feel bombarded with requests to participate. One way or the other, you (and your families) are the primary stakeholders in ALS research. It is critical that health professionals/researchers learn from your experiences and reflections about research and research participation. Please share your experience with research conducted both within the clinic and possibly at other sites or online.

People hear about research in different ways. In the poll below please indicate how you have heard about research opportunities.
Please select all the options that are applicable for you. If we've missed something, please click "reply: and share where else you've heard about opportunities to participate in research.

How have you heard about ALS research opportunities?
(after you make your selection, you will see the group results)

- [ ] In ALS clinic
- [ ] In a naturopathic clinic
- [ ] On the internet
- [ ] From other people
- [ ] From the ALS Society
- [ ] In a support group
- [ ] From a different source

4.2 [Topic 4, week 1]

I know that you're participating in this focus group! That's great!

1. Have you participated in other ALS-related research? Here are some questions to consider as you share your thoughts and experiences:
   - What kind of study/studies have you participated in? For example, drug trials? Interview studies? Surveys? Market research (research conducted by a commercial company; may provide payment for participation)? Other?
   - What did the study involve?
   - Have you heard of of ALS-related research opportunities, but been uninterested in participating? Please describe what makes you interested or not interested?
   - Have you heard of ALS-related research opportunities, but not been eligible to participate? Please share how you or your loved one felt about being ineligible
   - What kinds of studies are you most interested in?
2. In the exercise below, we've listed some reasons you might have for participating in a research study. Please rate from 1 to 7 what might inspire you. Click and drag the 'reasons for participating' over to the right and put in ranked order.
   - Number 1 should be the most important reason for you.
   - Number 7 should be the least important reason for you.
   - If you feel that some reasons are equally important to you, rate them the best you can and then use "reply" to share your thoughts.
   - If there is something else that inspires you to participate in research, please share that reason when you reply to this question.

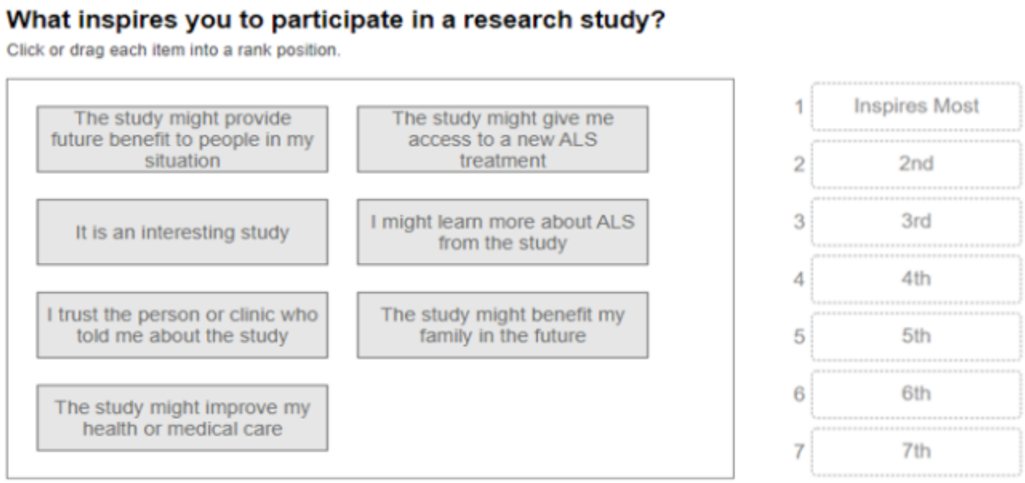

<u>**4.3** [Topic 4, week 2]</u>

All of us dream of the day when there will be good treatments and maybe even a cure for ALS. In the meantime, we do sometimes hear about ALS therapies or treatments that are 'in the pipeline'. These are new drugs or treatments that are at some stage of research or in the process of becoming available for use. These might be early clinical trials that we read about online. Sometimes we read or hear press releases from drug companies. And sometimes we know of drugs that are caught up in regulatory approval and are available in another country but not (yet?) in Canada.

1. **Please share how you think about and deal with information about ALS therapies or treatments that are in the 'pipeline.' Here are some questions to consider as you share your experience:**
    - Have you heard of a new drug or treatment that is 'in the pipeline' somewhere? Where did you hear about this?
      How do you respond if/when you hear about emerging ALS treatments? Do you look for further information? What are the important things you want to know?
    - How do you decide if information about a new therapy or treatment is trustworthy?
    - In an ideal world, what would be the best way to hear about therapies or treatments that are still in the research or approval 'pipeline'

2. **Even after there are positive results in the research setting, getting new treatments into people's hands can be a long process. Edaravone (Radicava) is a good example of this in Canada. Who do you think should be involved in this process in Canada? And how should they be involved?**
    - Federal government
    - Provincial health agencies
    - Insurance companies
    - ALS Society

- Other?

4.4 [Topic 4, week 2]

We've talked about looking for information about therapies or treatments available outside 'mainstream' health systems (often referred to as 'complementary and alternative treatments' or CAM). It's not uncommon for people to participate or think about participating in these sorts of therapies.

1. Would you like your 'mainstream' physicians, and in particular the doctors at the ALS clinics, to be involved with alternative and complementary therapies? If so, in what way should they be involved? Do you view information, support, or therapies from naturopathic practitioners as working *alongside* 'mainstream' physicians or as an *alternative* to the mainstream health system?

2. Have you participated, or are you planning to participate, in complementary and alternative therapies, or treatments available outside the mainstream medical clinics? These might include, for example:
    - Lifestyle changes, changes to diet, herbal or vitamin supplements, acupuncture, etc
    - Stem cell treatments, or other treatments that have not received regulatory approval
    - Interventions that involved travel to practitioners or clinics outside your city or province
    - Treatments or therapies not covered by the public health system

3. What motivates or persuades you to participate or *not* participate in these therapies/treatments? Have you had conversations with other people about these decisions? (e.g. family? friends? health professionals? naturopathic practitioner?)

5. Planning for future medical care

5.1 [Topic 5, week 1]

In the next few weeks we're going to discuss planning for future medical care. This could be a difficult area for some people. But the people with ALS who reviewed the focus group questions, as well as people in other focus groups, have told us that they value a chance to have open and honest discussion about this topic. Please remember: you don't have to answer every question. Also, share only what you feel comfortable discussing. We want to learn from your experiences and thoughts. We want to help health professionals do a better job when they are talking with people about advanced care planning.

Here are some advanced care planning topics that people might think about or talk about with a health care professional. Are you familiar with these topics? Please fill in the poll.

(NOTE: If you are having issue and cannot see it below, you can click on the 'Open In A New Window' button below. When you click on this button, a new window will appear for you to answer in. Once you have completed the exercise please ensure that you have clicked the 'Continue' button before closing the window to return to this area of the project.)

No, I am not familiar with this	I have heard of this	I know a little about this	Yes, I am familiar with this	**Topics related to advanced care planning**
				**Goals of care** (a medical order that lets the health care team know your wishes for care and for medical interventions)
				**Advance directive or 'living will'** (a legal document which specifies what health-related decisions should be made if a person is no longer able to make decisions for themselves)
				**Surrogate decision maker or "health care proxy"** (a person who will make personal care or health decisions for a someone who is no longer able to make decisions for themselves)
				**Palliative care** (medical care that focuses on quality of life and symptom control)
				**Withdrawn of care with palliative sedation** (stopping interventions that are sustaining life (e.g. ventilation) and providing medication to achieve physical comfort until natural death)
				**Medical assistance in dying** (a health professional either administers a drug that will cause death or provides a drug for an individual to take themselves to cause death)

5.2 [Topic 5, week 1]

Please share, as you feel comfortable, your experiences and thoughts about the following aspects of advanced care planning.

1. Have you talked to a health care professional about advanced care?
    - If *YES*: Please share how you experienced talking to a health care professional about advanced care.
      Here are some questions to guide you:
        - What triggered conversation about advanced care?
        - Who started the conversation?
        - Describe how the conversation was helpful and/or supportive, or how it was *not* helpful/supportive.
        - What aspects of advanced care planning have you talked about with your health professional?
        - Do you feel adequately informed about this topic?
        - Was there something that you wanted or needed the healthcare professional to say/do that they did not say/do?
    - *If NO*: Do you want to talk with a health care professional about this topic? Who should start this conversation? Are there aspects of advanced care that you want or need to learn about?

2. Please describe what makes you comfortable or *not* comfortable when talking with health professionals about advanced care planning. Are there particular topics you

find difficult to talk about with health care professionals? Is there a different person you prefer to talk to (for example, a spiritual counselor) about advanced care planning or end of life decisions?

5.3 [Topic 5, week 2]

Our beliefs and backgrounds can be really important to who we are as individuals. They can also play a big role in how we face the experiences that life brings us. Here are a few questions about those factors.

Please share how your world view influences you as you approach conversations and decisions about future care and/or end of life decisions? For example, what role does spirituality or religion play for you? What role does your culture, family, life experience, and/or personal philosophy play in these conversations and decisions?

5.4 [Topic 5, week 2]

Because research has not yet found a cure for ALS, discussion of "prognosis" - the anticipated or likely disease course - can be important.

We are interested in how you think *prognosis* should be discussed with people living with ALS and their families.

Here are some questions to consider as you share your experience and thoughts:

- What is your experience talking with health professionals about the ALS prognosis?
- How do you think health professionals should discuss prognosis with people who have been diagnosed with ALS and with their families?
- Would it be helpful to talk about individual, personal prognosis instead of the general prognosis for people with ALS? How would this be helpful, or *not* helpful, for you? How is understanding of general prognosis helpful, or *not* helpful, to you?

6. Conversations about death and dying

6.1 [Topic 6, week 1]

Advanced care planning may bring conversations about death and dying. Health care professionals need to understand how people living with ALS want to learn and talk about these topics. This is important because health professionals are not always very good at talking about death and dying. Please share and discuss with the group as you feel comfortable.

1. What does a 'good death' or 'peaceful death' mean for you? What comes to mind when you think about this from physical, emotional, relationship and/or spiritual

perspectives? Do you have thoughts about how you want it to be? For example: Where would it happen? Who would be there?

2. The most common approaches to peaceful dying, for people with ALS, are listed below. Have you discussed any of these approaches with a health care professional?
3. For people who are on life-sustaining treatments (for example, using BIPAP much of the day and night) AND wish to bring life to a close, 'palliative sedation for the withdrawal of treatment' may be a less bureaucratic option than is MAiD. A person may make this decision with their doctor and there is no formal approval process or wait time.

If you reached the point where you wanted to bring life to a close,

- Would you be open to 'withdrawal of treatment with palliative sedation'? To MAiD?
- Why might you lean towards one or the other, if at all?
- Do you think you would be open to whichever approach suited your situation at that time?

Palliative care
- Medical care focusing on quality of life and symptom control in the absence of cure.
- Medical care to ensure physical comfort until natural death.

Palliative sedation for the withdrawal of treatment
- Life-sustaining interventions are stopped (e.g. ventilation) AND medication is given to ensure physical comfort until natural death.
- Decision-making involves the patient and doctor (and loved ones). No wait period required.

Medical assistance in dying (MAiD)
- A health professional either administers a drug that will cause death or provides a drug for an individual to take themselves to cause death.
- Approval by two doctors required and a 10 day wait period.

6.2 [Topic 6, week 1]

Everyone has different things that they want or do not want to talk about. Sometimes this can be a challenge.

1. Please describe if you have *wanted* to discuss death and dying with someone - a health professional or perhaps a loved one - but felt uncomfortable to do so. What would you like to talk about?

2. Has anyone – a health professional or someone else – every started an *unwanted* conversation with you about death and dying? What was it about the conversation that made you uncomfortable or that you did not like? How did you respond?

6.3 [Topic 6, week 2]

In June 2016, the Parliament of Canada passed legislation allowing eligible Canadians to request medical assistance in dying (sometimes called 'MAiD,' or voluntary assistance in dying). We want to better understand what this means to you. We welcome all perspectives on this topic.

We want to hear from you if you are NOT in favor of this new legislation, if you ARE in favor of it, and if you aren't sure what you think about it. We want to learn from everyone. Please share and discuss with the group as you feel comfortable.

What are your thoughts about medical assistance in dying? Please describe what influences your thoughts and views about this. Here are some questions to consider as you share your experience and thoughts:

- How do you think this Canadian legislation affects people with ALS?
- How do you think it influences the families and friends of people with ALS?
- How do you think it influences other people?
- Do you think health care professionals should start a conversation about MAiD with people living with ALS? Why or why not?

<u>**6.4** [Topic 6, week 2]</u>

There's been quite a bit of media coverage for medical assistance in dying (MAiD).

What do you think about the media coverage of medical assistance in dying? How do you think media coverage might affect other people? Please describe *if* and *how* media discussion has affected you. Please describe *if* and *how* media coverage has influenced your views on medical assistance in dying.

7. Improving ALS Communication and support

<u>**7.1** [Topic 7, week 1]</u>

Thank-you for making it this far with the group! An important goal of this study is to help health care professionals talk with people living with ALS and their families/caregivers. Here's a chance to discuss your ideas about communication and how communication can be improved.

1. Below are some of the things we heard from the different focus groups about 'good communication.' What have we missed? Do any of these suggestions *not* quite fit your experience? What would you suggest instead?

   Focus group suggestions for health care professionals:

   a. Be matter of fact and share the best information available while showing empathy and compassionate. (No beating around the bush!)
   b. The person with ALS is in the driver seat. They have the final say in decisions and should have all the information needed to make the best decision for themselves.
   c. Be sensitive to individual circumstances and people's 'readiness' to have hard conversations.
   d. Be knowledgeable, open to learning about, and supportive of people's interest in the range of treatment options that are available within and beyond the healthcare system.

e. Be flexible and responsive to people's preferences. But be ready to raise (and return to) hard conversations if needed because of ALS progression.
f. People need time to process things. A soft early introduction to difficult topics followed by more direct communication as ALS symptoms make the conversation necessary can make adapting to changes easier.
g. Information that's given verbally, especially at the beginning, should be repeated in another format (written documents, etc.) for later reference.
h. Make space for supporting family members/caregivers. Ask them how they're doing! Check if they need support or information.

2. We've talked about the information you need as you navigate ALS. If you had every doctor, nurse, OT, PT, Speech therapist, personal care aid, ALS Society coordinator, etc. in the same room, what would be your *top recommendations for how they should provide information* to people with ALS and their families/caregivers? Are there any recommendations you would give to specific health professions?

3. Are there any topics or questions that you would like to discuss with a health professional, but find it difficult to do so? Why are these topics difficult to discuss with a health professional? (*We've touched on this before. If you don't have anything to add, that's OK!*)

7.2 [Topic 7, week 1]

A secondary goal of the focus groups has been to 'pilot' test the idea of online support groups for people living with ALS. Here are a few questions about the online aspect of this group, and support groups in general.

1. Do you think *online* support groups that allow people living with ALS to connect and share information are helpful? Why, or why not?

2. Please share *how* or *if* you think online support groups would be helpful for family members/caregivers of people living with ALS.

3. Sometimes people in the ALS community shy away from peer support because of anticipated losses. What are your thoughts about peer support in the face of these difficult realities? How might you encourage engaging in peer support, or would you at all? (This question was touched on earlier in this focus group - if you have nothing further to say, that's OK. If you want to add or emphasize something, please feel free to do so!).

7.3 [Topic 7, week 2]

Because of the COVID 19 situation, some interactions that used to take place face-to-face are now taking place online or via telephone. On one hand, this can isolate many of us in our homes.

On the other, communicating virtually or phone meetings may reduce the time and energy spent on travel.

1. Have you had virtual appointments, email exchanges or telephone conversations that have taken the place of face-to-face interactions with health professionals? What has this been like for you?
    - Have these forms of interaction taken the place of face-to-face interaction with people at the ALS Society? What has that been like?

2. Do you have concerns about virtual or other 'distant' interactions with health professionals? Do you see advantages?

3. Can you see a place for virtual appointments when in-person clinic (or doctor) visits are an option again? Can you see a place for virtual appointments with any other members of the health care team (e.g. social workers, dietitians, etc)?

7.4 [Topic 7, week 2]

Communication isn't just about what is said. It's also about the way information is given and/or supported.

Here are some recommendations we heard from the focus groups.
Which of these do you think people diagnosed with ALS today, and their families, would find most valuable? What have we missed? What would you suggest instead?

1. Make follow up calls within a couple days of an ALS diagnosis or other critical appointments.
2. Provide contact information (emails? telephone numbers?) so people can contact someone on the health care team at important moments of need.
3. Provide writing materials so that people can make notes at appointments if they like.
4. Provide written material or handouts about the topics discussed at appointments.
5. Early after diagnosis, provide written, reference material with an overview of ALS progression (e.g. changes in speech, swallowing, nutritional management, mobility and breathing). Make sure to emphasize that progression varies.
6. Highlight the importance of and provide written information about the ALS Society.
7. Make sure people know about the 'ALS manual' from the ALS Society
8. Recommend reliable websites for ALS information.

7.5 [Topic 7, week 2]

Many of you have described clinic visits as being physically, mentally and/or emotionally exhausting. While our focus has been on communication, that's only one part of a broader relationship between you and the healthcare team.

What do you think is most important for your health care professionals to know in order to improve your experience at the clinic?

<u>**7.6** [Topic 7, week 2]</u>

We'll have some final thoughts and information next week, <u>as well as an extra, optional question topic</u> :)

In the meantime, if you have any feedback about the focus group itself, we would really like to hear it. And please, let us know <u>if</u> and <u>how</u> you would like to learn about the findings and outcomes of this focus group study (poll below).

Thoughts about the focus group study. *If you'd like to send a response in a private message, start typing in the 'reply' space and then you'll see a small checkbox with the option to "Make Private", or use the slider on the app.*

Some questions to consider:

- What have you liked or *not* liked about participating in this online, focus group study?
- Were there other topics that you would have liked to discuss?
- What do you think about the length of the focus group?
- How could we have made the focus group better?

Future contact. If you would like to receive updates by email, we will use the email address that you are using for this study. If you want to use a different email address, send your updated contact information to us at als.talk@ualberta.ca.

How you would like to stay in touch?

○ I don't want to receive findings or outcomes of the study - thanks!

○ I would like findings or outcomes to be posted on the ALS Talk website. I will look at them when I choose.

○ I would like to receive an email about findings or outcomes posted on the ALS.Talk website.

Comments and feedback: You have 3 weeks to make final comments

Optional topics

Optional topic: Observational research & data sharing

[All questions posted; participants given 2 weeks to engage with this optional topic]

1 [Optional topic, week 1]

*In 2019, Canadians living with ALS sent 2378 letters to the federal government to petition for research funds to support a proposed research study called CAPTURE ALS. We're going to use this proposed study as a 'case study' to illustrate Discussion topic 8.**

The goal of CAPTURE ALS is to 'paint' a detailed picture of ALS. Medical data gathered from people with ALS and healthy volunteers (including interested family members) will be used to help understand the causes of ALS, understand the different ways that ALS affects people, and identify new treatments.

Participants will undergo a variety of assessments, repeated over time. For example, they will answer questions about symptoms, undergo brain scans, cognitive and speech testing, and have blood, urine and spinal fluid collected. Participants will interact with medical staff and researchers who will conduct assessments at intervals similar to the intervals between clinic visits. Anonymous medical data from participants will be securely stored in a database.

*NOTE: We are not recruiting for this study. We want to learn more about what you think about studies that do *not* involve a possible treatment for participants.

Your answers in Discussion Topic 4 showed that people are highly motivated by research that might improve health or well-being, or give access to a new treatment. Your answers also suggest interest in research that explores ALS cause(s) and in having better data about people with ALS. People also shared frustration about the tight rules for drug trials (clinical trials) and not being eligible to participate.

1. Studies that don't involve a direct treatment are called 'observational studies.' More people are eligible to participate in these studies. What do you think about participation in a study like CAPTURE ALS that does *not* include a treatment possibility? Here are some questions to guide your thoughts:
    - What might make you interested in participating in a study that *does not* involve a treatment?
    - How much effort would you feel was worthwhile if you were participating in a study that advanced knowledge about ALS (e.g. the cause of ALS) but had no impact on your disease course or the disease course of a loved one?
    - How would you feel about being part of a long term study that continued throughout the course of ALS?

2. What aspects of the case study do you find interesting? What would you want to know if you were considering participation?

2 [Optional topic]

We want to better understand some of the practicalities that influence people who think about participating in research.

1. Some people have shared how visits to the ALS clinic can be overwhelming and/or exhausting.
   - What is the best way for the ALS Clinic to tell people about research studies they might participate in?
   - When is the best time to approach people? (For example, how early in the ALS journey?)
   - Who should share information about studies?

2. How should the ALS Society tell people with ALS and their families about studies that are looking for participants? Share the information on their website? Send out emails? Newsletter? Personal contact?

3. What might an observational study, like our case study, 'give back' that would be valuable to participants? Choose as many options in the poll as are applicable for you. If you choose "something else", write a response and share what this might be.

- ☐ Copies of your brain imaging
- ☐ Access to genetic testing results and counselling
- ☐ Copies of your data (e.g. strength tests, breathing scores, etc)
- ☐ Your results compared to average results
- ☐ Regular interaction with health professionals and researchers
- ☐ Something else?

3 [Optional topic]

You've hung in for this focus group study! It's been a number of months and we're super grateful.
We want to understand what people living with ALS and families/caregivers think about participating in a long term study like the case study, CAPTURE ALS.

What would encourage you to continue participation in a study that requires collection of medical information for more than a year?
(Choose as many options as are applicable for you. If you choose "something else", write a response and share what this might be.)

- ☐ Costs covered when visits to the research centre are required to complete assessments
- ☐ Cost of overnight hotel covered when travel to research centre is needed to complete assessments
- ☐ Assessments in the home, where possible
- ☐ Feedback about the study while the study is in progress
- ☐ Opportunity to interact with other people in the study in an online forum
- ☐ Opportunities for Q and A with researchers
- ☐ Opportunities to hear researchers talking about research results from studies done with your data
- ☐ Opportunities to provide feedback on your experience participating in the study
- ☐ Something else?

4 [Optional topic]

Some assessments in our case study (CAPTURE ALS) may be optional, for example, in-depth neuro-psychological assessment or nerve-testing.

What would be important to you if you were considering participation in optional assessments?
(Choose as many options as are applicable for you. If you choose "something else", write a response and share what this might be.)

- ☐ How the timing of the optional assessment fits in with my other life commitments
- ☐ Amount of time and energy involved in the optional assessment
- ☐ Whether the optional assessment could be done in my home or community
- ☐ My relationship with researchers and research staff
- ☐ Receiving information about my assessment results
- ☐ If the additional study caused any physical discomfort
- ☐ Something else?

5 [Optional topic]

One of the goals of the case study is to develop a database containing anonymous information and samples from participants. This database could be securely shared with other researchers.

Sharing data with qualified researchers outside the original research team will increase the number of researchers investigating ALS cause(s) and variability, and working towards treatments.

It's critical for researchers to understand what people living with ALS and their families/caregivers think about sharing anonymous medical information.

1. What do you think about giving permission and allowing your anonymous medical information and/or biosamples to be shared with researchers in Canada? How do you feel about sharing this anonymous information with researchers from other countries? What questions would you want answered before you might give this permission?
2. What do you think about sharing your anonymous medical information and biosamples with profit-based pharmaceutical companies working on ALS drug development? What questions would you want answered before you might give this permission?

Optional topic: COVID-19

[Optional topic posted March 21, 2020 (AB, ON) and April 3rd, 2020 (BC, QC/NS/NB). Available for input for the duration of the focus groups.]

In light of the extraordinary circumstances that we find ourselves in, we wanted to give you a space to talk about Coronavirus (Covid 19) and how it has impacted you. We are aware that aspects of this virus are concerning. A few people in the focus groups have commented on Covid-19 in other topic discussions. That's perfectly fine! Please feel free to ignore this option discussion thread, or read and add comments as you wish.

We are interested to know how you have learned about this new virus and how you are protecting yourselves.

Here are a few questions to guide your comments and discussion.

- How did you first learn about Coronavirus? At what point did you begin to consider its potential impact on you and your loved ones?
- How have you gone about learning about coronavirus?
  - Have you relied on the news media? Explored online information? Contacted a health professional? Talked to someone at the ALS Society? Something else?
  - What has been helpful, or *not* helpful, for you?
- What are your primary concerns related to coronavirus? Please describe if you've been able to talk to someone about these concerns.
- How is, or how *should* the ALS clinic, your doctor (other health professionals), or the ALS Society support people living with ALS during this time?
- What changes have you made in your own life to adapt to the potential dangers of the virus? Are you planning to make future changes?
