# Supplementary material for: Asynchronous online focus groups for research with people living with amyotrophic lateral sclerosis and family caregivers: usefulness, acceptability and lessons learned
Source: BMC Med Res Methodol. 2023 Oct 6;23:222. doi: 10.1186/s12874-023-02051-y (PMC10557269; doi:10.1186/s12874-023-02051-y)
Supplement: Supplementary file 2 — Additional file 2. [file 12874_2023_2051_MOESM2_ESM.pdf]

Additional file 2 for "Asynchronous online focus groups for research with people living with amyotrophic lateral sclerosis and family caregivers: Usefulness, acceptability and lessons learned"

Shelagh K. Genuis[1], Westerly Luth[1], Garnette Weber[2], Tania Bubela[3], Wendy S. Johnston[1]*

* Correspondence: wendyj@ualberta.ca
[1]Division of Neurology, Department of Medicine, University of Alberta, Edmonton, Alberta Canada
[2]itracks, Saskatoon, Saskatchewan, Canada
[3] Faculty of Health Sciences, Simon Fraser University, Burnaby, British Columbia, Canada
Full list of author information is available at the end of the article

Technology used by participants
(Asynchronous online focus groups for people living with ALS)

Technology used	N=100
**Device**	
Desktop	78
Smartphone	18
Tablet	4
**Browser**	
Chrome	59
Safari	36
Firefox	4
MSIE 11	1
**Operating System**	
Windows NT 4.0	40
Mac OSx	30
Ios (Iphone/iPad)	16
Windows 8	5
Android mobile OS	5
Linux/Unix	4
Windows 7	4
Windows Vista	1
